# The circular RNA circSLC7A11 functions as a mir-330-3p sponge to accelerate hepatocellular carcinoma progression by regulating cyclin-dependent kinase 1 expression

**DOI:** 10.1186/s12935-021-02351-7

**Published:** 2021-11-29

**Authors:** Yu Huang, Wenhao Ge, Yuan Ding, Lufei Zhang, Jiarong Zhou, Yang Kong, Bijun Cui, Bingqiang Gao, Xiaohui Qian, Weilin Wang

**Affiliations:** 1 Department of Hepatobiliary and Pancreatic Surgery, The Second Affiliated Hospital, Zhejiang University School of Medicine, No. 88 Jiefang Road, Hangzhou, 310009 Zhejiang China; 2 Key Laboratory of Precision Diagnosis and Treatment for Hepatobiliary and Pancreatic Tumor of Zhejiang Province, Hangzhou, 310009 Zhejiang China; 3 Research Center of Diagnosis and Treatment Technology for Hepatocellular Carcinoma of Zhejiang Province, 310009 Hangzhou, China Zhejiang; 4 Clinical Medicine Innovation Center of Precision Diagnosis and Treatment for Hepatobiliary and Pancreatic, Disease of Zhejiang University, Hangzhou, 310009 Zhejiang China; 5 Clinical Research Center of Hepatobiliary and Pancreatic Diseases of Zhejiang Province, Zhejiang Hangzhou, 310009 China; 6 Zhejiang University Cancer Center, Hangzhou, 310009 Zhejiang China

**Keywords:** circRNA, Competing endogenous RNA, Hepatocellular carcinogenesis, Cell cycle, CDK1

## Abstract

**Background:**

Circular RNAs (circRNAs), which are endogenous non-coding RNAs, are associated with various biological processes including development, homeostatic maintenance, and pathological responses. Accumulating evidence has implicated non-coding RNAs in cancer progression, and the role of circRNAs in particular has drawn wide attention. However, circRNA expression patterns and functions in hepatocellular carcinoma (HCC) remain poorly understood.

**Methods:**

CircRNA sequencing was performed to screen differentially expressed circRNAs in HCC. Northern blotting, quantitative real-time polymerase chain reaction, nucleocytoplasmic fractionation, and fluorescence in situ hybridization analyses were conducted to evaluate the expression and localization of circSLC7A11 in HCC tissues and cells. CircSLC7A11 expression levels were modified in cultured HCC cell lines to explore the association between the expression of circSLC7A11 and the malignant behavior of these cells using several cell-based assays. The modified cells were implanted into immunocompetent nude mice to assess tumor growth and metastasis in vivo. We applied bioinformatics methods, RNA pulldown, RNA immunoprecipitation, and luciferase reporter assays to explore the mechanisms of circSLC7A11 in HCC.

**Results:**

CircSLC7A11 (hsa_circ_0070975) was conserved and dramatically overexpressed in HCC tissues and cells. HCC patients showing high circSLC7A11 expression had worse prognoses. Our in vitro and in vivo experiments showed that circSLC7A11 markedly accelerated HCC progression and metastasis through the circSLC7A11/miR-330-3p/CDK1 axis.

**Conclusions:**

The acceleration of HCC progression and metastasis by circSLC7A11 through the circSLC7A11/miR-330-3p/CDK1 axis suggests that circSLC7A11 is a potential novel diagnostic and therapeutic target for HCC treatment.

**Supplementary Information:**

The online version contains supplementary material available at 10.1186/s12935-021-02351-7.

## Background

Hepatocellular carcinoma (HCC) is among the most common cancers worldwide, and has a consistently poor prognosis [1]. Histopathologic diversity and distinct histologic subtypes of HCC are well-recognized [2]. Despite advances in surgical resection, adjuvant therapy and liver transplantation, the prognosis of HCC patients remains unsatisfactory, due to late diagnosis and the high risk of potential recurrence and metastasis [3]. Other non-surgical interventions, including trans-arterial chemoembolization (TACE), radiofrequency ablation (RFA), and systematic sorafenib administration, exert only weak therapeutic effects in advanced-stage HCC patients [4]. The recent emergence of cancer immunosurveillance technology has led to the development of immune checkpoint blockades in cancer therapy [5]. However, most HCC patients treated with these new agents have shown weak clinical responses, sometimes even developing drug resistance [6]. With the concept of precision medicine and development of organoid culture, whole-exosome sequencing, mass spectrometry-based proteomics and metabolomics and single cell analysis, diverse precise molecular subtypes of HCC were proposed based on the genomes, transcriptomes, proteomes and metabolomes mutation. For instance, Calderaro et al. reported CTNNB1 (40%) and TP53 (21%) mutations were mutually exclusive and defined two major groups of HCC characterized by distinct phenotypes, which is beneficial for guiding different therapeutic strategies to some extent [7]. Another research revealed that, by clustering immune cells in the HCC microenvironment, HCC subtypes were identified as immunocompetent, immunodeficient, and immunosuppressive features, which was meaningful for selecting patients suitable for developing local immunotherapy treatment [8]. Therefore, a thorough investigation of the mechanisms associated with HCC progression and recurrence would contribute to distinguish of molecular subtypes and development of new therapeutic approaches for HCC patients [9].

Accumulating evidence has closely implicated non-coding RNAs in cancer progression [10]. CircRNAs, which are endogenous noncoding RNAs within eukaryotes, are characterized by a covalent closed-loop structure with a canonical splicing junction site, no 5’-cap structure, and a 3’-poly A tail. CircRNAs were once regarded as meaningless splicing error by-products [11]; however, the development of high-throughput sequencing and bioinformatic technologies has revealed that circRNAs are pervasive and generally stable, and show variable expression among different cells and tissues, suggesting that circRNAs possess specific biological functions [12]. More recently, circRNAs were demonstrated to exert diverse functions, acting as miRNA sponges, binding to proteins, and participating in protein translation [13, 14]. CircRNAs also appear to function as mediators in cancers via various mechanisms [15, 16]. For example, circDONSON promoted the progression of gastric cancer via NURF complex-dependent activation of SOX4 signaling [17]. CircFoxo3 was reported to induce cell apoptosis by increasing Foxo3 activity [18]. Other studies have revealed that circRNAs such as cSMARCA5, circ-CDYL, circMAT2B, and circASAP1 strongly impact HCC progression [19–22]. Thus, in-depth research on the functions of circRNAs and their molecular mechanisms in HCC pathogenesis have contributed significantly to elucidating the clinical value of circRNAs.

MicroRNAs (miRNAs) are ubiquitous, conserved, small noncoding RNAs that act as negative regulators to suppress the expression of target genes [23]. Competing endogenous RNAs (ceRNAs) are transcripts that sponge target miRNAs, mediating post-transcriptional regulation via competitive binding [24]. Recent studies have reported that circRNAs become ceRNAs by acting as miRNA sponges, binding to miRNA response elements (MREs) to repress functions [25]. For example, ciRS-7 was found to act as an efficient miRNA sponge [13]. Thus, the circRNA–miRNA regulatory network is becoming a cancer research hotspot.

HCC is largely the result of uncontrolled cellular proliferation following a series of disruptions of normal cell cycle regulatory checkpoints [26]. Cyclin-dependent kinase 1 (CDK1) is a canonical cell cycle regulatory checkpoint that participates in cell proliferation and transcription regulation [27]; it is essential for cell cycle progression to the M phase, regulating the S–G_2_ and G_2_–M transitions in association with cyclin A and B, respectively. Previous studies have reported that CDK1 overexpression is associated with clinicopathological features and poor prognosis of HCC patients. Cell cycle progression has been blocked at the G_2_–M phase transition in HCC cells through CDK1 downregulation, both *in vitro* and *in vivo* [28, 29]. CDK1 inhibitors such as flavopiridol have shown synergistic antitumor effects through increasing apoptosis and decreasing proliferation among tumor cells when combined with conventional chemotherapeutics [30, 31]. However, the upstream mechanism of CDK1 dysregulation remains largely unknown in HCC. A deeper understanding of the underlying mechanisms of action of CDK1 in HCC pathogenesis is vital for its diagnosis and therapy.

In this study, three pairs of HCC tissues of which pathological grade including well/moderate/advanced differentiation, along with their adjacent normal tissues, were used for RNA sequencing and we identified a novel HCC-related circRNA, circSLC7A11, originating from exons 8 and 9 of the SLC7A11 gene and with a circBase ID of hsa_circ_0070975. To determine the function and underlying mechanism of circSLC7A11, we assessed its clinical significance in HCC patients, and modified its expression in HCC cell lines to explore the association between the expression of circSLC7A11 and the malignant behavior of HCC cells. Next, we implanted stably transfected sh-circSLC7A11 cells into immunocompetent nude mice to assess the effects of circSLC7A11 on tumor progression and metastasis *in vivo*. Our findings could aid prediction of the prognosis of HCC and provide new insight into potential therapeutic targets.

## Methods

### Patient tissue samples and cell lines

Human tumor samples were obtained from patients with HCC who had undergone curative resection from 2015 to 2016 at the Second Affiliated Hospital, Zhejiang University School of Medicine, Hangzhou, China. This study was approved by the Ethics Committee of the Second Affiliated Hospital, Zhejiang University School of Medicine (No.2021-236) and conformed to the principles of Declaration of Helsinki. Written informed consent was obtained from all patients.

The human HCC cell lines HepG2, Hep3B, HCCLM3, Huh7, and MHCC97H, and the immortalized hepatocyte cell line WRL68 were purchased from Shanghai Institutes of Biological Sciences, Chinese Academy of Sciences. All cells were maintained in high-glucose Dulbecco’s modified Eagle’s medium (DMEM; Cat# No. 8,121,393; Gibco) supplemented with 10% fetal bovine serum (FBS; Cat# No. 10,091,148; Gibco) and 1% penicillin/streptomycin (Cat# No.15140-122; Gibco) in an incubator at 37  °C with a humidified atmosphere containing 5% CO_2_, and passaged following standard cell culture techniques.

### Total RNA extraction and quantitative real-time polymerase reaction (qRT-PCR)

Total RNAs of HCC tissues/cells and paired normal tissues/cells were extracted using TRIzol reagent (Cat# No. 15,506,018; Invitrogen, Carlsbad, CA, USA). The concentration of RNA was detected using a Nanodrop 2000 system (Thermo Fisher Scientific). For circRNA and mRNA, reverse transcription was conducted using the HiScript II Q RT SuperMix for qPCR (Cat# No. R223-01; Vazyme Biotech, Nanjing, China) with random primers. For miRNA, reverse transcription was conducted using the HiScript II Q Select RT SuperMix (Cat# No. R233-01; Vazyme Biotech) with specific stem-loop primers. cDNA amplification was performed using ChamQ Universal SYBR qPCR Master Mix (Cat# No. Q711-03; Vazyme Biotech) with an ABI Prism 7500 sequence detection system (Applied Biosystems, CA, USA). Relative RNA expression levels were normalized to those of internal controls (GAPDH and U6) using the 2^−ΔΔCT^ method. All primers were designed by Tsingke Biological Technology (Beijing, China) and are listed in Additional file 1: Table S1.

### Nucleic acid electrophoresis

The PCR products were subjected to nucleic acid electrophoresis using 2% agarose gel with 0.5% TAE running buffer at 100 V for 40 min. We used DL500 DNA Marker (50–500 bp) (Cat# No. 3590A; TaKaRa, Osaka, Japan) to observe the bands under ultraviolet irradiation.

### Oligonucleotide transfection

HCC cells were seeded and cultured to 50–60% confluence before transfection. Si-circSLC7A11 and the negative control were designed and synthesized by RiboBio (Guangzhou, China). The miR-330-3p mimics, inhibitor, and negative control were synthesized by GenePharma (Cat# No. B03001; Shanghai, China). Lipofectamine 3000 reagent (Cat# No. L3000015; Invitrogen) was used as a transfection medium. All oligonucleotide sequences are listed in Additional file 2: Table S2.

### Overexpression vector construction and transfection

The pCDH-ciR vector used in our research was synthesized by RiboBio (Guangzhou, China). To construct the overexpression vector of circSLC7A11, full-length circSLC7A11 cDNA was cloned into the pCDH-ciR vector; empty vector without the circSLC7A11 sequence was used as a negative control.

### RNase R treatment

RNAs (10 µg) extracted from HCCLM3 and Hep3B cells were treated with RNase R (3 U/µg, Cat# No. R0301; Geneseed, Guangzhou, China) and incubated for 30 min at 37  °C. The treated RNAs were reverse-transcribed with divergent or convergent primer and detected by qRT-PCR or nucleic acid electrophoresis.

### Western blot analysis

Tissues or cells were lysed in radio immunoprecipitation assay (RIPA) lysis buffer (No. P0013B; Beyotime, Shanghai, China) containing 1 mM phenylmethylsulfonyl fluoride (PMSF, Cat# No. ST505; Beyotime, Shanghai, China) for 30 min on ice. After centrifugation (15,000 ×* g*, 4  °C, 15 min), the supernatant was collected. A bicinchoninic acid (BCA) protein assay kit (Cat# No. 23,225; Thermo Fisher Scientific) was used to measure protein concentrations and all protein samples were normalized to 2 μg/ul. For electrophoresis, equal amounts of denatured proteins (20ug) were separated on 4–12% NuPAGE Bis-Tris Gel (Cat# No. M00653; GenScript, Piscataway, NJ, USA) and then transferred onto 0.45-µm polyvinylidene fluoride (PVDF) membranes (Cat# No. ipvh00010; Millipore, Burlington, MA, USA). The membranes were blocked in 5% non-fat milk in TBST and incubated with the indicated primary antibodies overnight at 4  °C. Then, the membranes were incubated with horseradish peroxidase (HRP)-conjugated secondary antibodies (1:3000) for 1 h at room temperature, followed by detection using an imaging system (Bio-Rad, Hercules, CA, USA). The primary antibodies and dilution of antibodies used in this experiment are listed in Additional file 3: Table S3.

### Cell counting Kit-8 proliferation assay

Cell proliferation was evaluated using the Cell Counting Kit-8 (Cat# No. CK04; Dojindo Corp., Japan). A total of 4,000 transfected cells were seeded in each well of a 96-well plate. At the indicated times (0, 24, 48, and 72 h), 10 µL of CCK-8 reagent was added directly to the culture medium. Then, the cells were incubated for 2 h at 37 °C, and the optional density at 450 nm (OD_450_) was measured using a microplate reader (BioTek Instruments, USA).

### Colony formation assay

We seeded 1,000 transfected cells in each well of a 6-well plate for culturing at 37 °C in 5% CO_2_ for 14 days. After 14 days, cells were washed with PBS, fixed with 4% paraformaldehyde for 20 min, and stained with 0.1% crystal violet solution for another 20 min before the cell colonies were counted. Colonies containing 30 cells were selected and counted using light microscopy. The average number of colonies was determined from three independent experiments.

### 5-Ethynyl-20-deoxyuridine (EdU) incorporation assay

We performed an EdU assay to analyze the proliferative capacity of cells using the Cell-Light EdU DNA Cell Proliferation Kit (Cat# No. C10310; RiboBio, Guangzhou, China) following the manufacturer’s protocol. The cell lines were sequentially stained with Apollo Dye Solution and Hoechst 33,342, and EdU-positive cells were counted under a Leica DMi8 microscope (Leica, Germany).

### Wound-healing assay

HCC cells were seeded in a 6-well plate in medium with 10% FBS. After adherence, cells were scratched with a 200-µL pipette tip and replaced with serum-free medium. After washed with PBS, representative images were photographed 0 and 24 h after injury. The diminishing distance across the injury area was measured and normalized to the distance at 0 h. The data are expressed as relative migration rates.

### Immunohistochemical (IHC) analysis

Tissue samples were fixed in 4% paraformaldehyde embedded in paraffin and sectioned. The tissue sections were incubated with anti-CDK1, anti-E-cadherin, anti-N-cadherin, and anti-Ki-67 primary antibodies at 4 °C overnight and then incubated with HRP-conjugated secondary antibody. H Score (intensity of staining (4, 3, 2, 1) × % of positive cells staining with that intensity/100%) was used to analyze the quantification of IHC. The dilution of antibodies used in this experiment are listed in Additional file 3: Table S3.

### Fluorescence in situ hybridization (FISH)

A FISH assay was performed to determine the subcellular locations of circSLC7A11 and miR-330-3p. Briefly, circSLC7A11 probe and miR-330-3p probe were directly generated by Servicebio (A2061841, Wuhan, China) depending on their inverse complementary sequence, respectively. The corresponding probe sequences are listed in Additional file 2: Table S2. HCCLM3 cells were seeded in a cell slide and grown to 50-60% confluent at the time of fixation. After permeabilized with 0.25% Triton X-100 in PBS for 15 min and pre-hybridized in hybridization buffer (50% formamide, 10 mM Tris-HCl, 200 µg/ml yeast transfer RNA, 1⋅ Denhardt’s solution, 600 mM NaCl, 0.25% SDS, 1 mM EDTA, 10% dextran sulfate) for 1 h at 37 °C, the cell slide was heated to 65 °C for 5 min in hybridization buffer containing 3 nM digoxin-labeled miR-330-3p probe and 300 nM cy3-labeled circSLC7A11 probe and hybridization occurred at 42 °C overnight. The next day, after washed with 2 ⋅ Saline Sodium Citrate Buffer (SSC) for 10 min, 1⋅ SSC for 10 min twice and 0.5⋅ SSC for 10 min at 37 °C, nuclei were stained with 4’,6-diamidino-2-phenylindole (DAPI) for 10 min [32, 33]. The images were acquired using a laser scanning confocal microscope (LSM900; Zeiss, Germany).

### Biotin-coupled probe RNA pull-down assay

A biotinylated circSLC7A11 probe and negative control probe were synthesized by GenePharma (Shanghai, China). The probes were incubated with M280 streptavidin-coupled Dynabeads (Cat# No. 11205D; Invitrogen) at 25 °C for 2 h to generate probe-coated beads, which were then incubated with the cell lysates at 4 °C overnight. The beads were washed with wash buffer, and the RNA complexes were then purified with TRIzol reagent and subjected to PCR analysis. The probe sequences are listed in Additional file 2: Table S2.

### Dual luciferase reporter assay

The sequences of CDK1-3’UTR and their corresponding mutation were synthesized, cloned into luciferase reporter vector PGL3 (Genomeditech, Shanghai, China), respectively. All plasmids were co-transfected with miR-330-3p mimics or controls into HEK-293 T cells. Relative luciferase activity was detected using the Dual Luciferase Assay Kit (Cat# NO. E1910; Promega, Madison, USA).

### RNA immunoprecipitation (RIP) assay

RIP assay was performed using the Magna RIP RNA-Binding Protein Immunoprecipitation Kit (No.17-700; Millipore), according to the manufacturer’s instructions. The beads were washed and incubated with proteinase K to remove proteins. Finally, the concentration and quality of the purified RNA were measured and subsequently subjected to PCR analysis. The final expression level was normalized to the fold of the concentration between “IgG” and “Input”, and “AGO2” and “Input”, respectively, in a manner of “%Input”.

### Mouse models

All mice were housed under pathogen-free conditions in the animal facility of the Second Affiliated Hospital, Zhejiang University School of Medicine. Animal experiments were performed according to a protocol approved by the Ethics Committee of the Second Affiliated Hospital, Zhejiang University School of Medicine (No. 2021-44). Briefly, 6-week-old female BALB/c nude mice were purchased from the Shanghai Experimental Animal Center of the Chinese Academic of Sciences (Shanghai, China). HCCLM3 cells (5 × 10^6^) suspended in 150 µL PBS transfected with sh-circSLC7A11 or negative control were subcutaneously injected into the left flank of each mouse. The tumor volume was measured every 7 days using a caliper and calculated as (length × width^2^)/2. After 4 weeks, mice were sacrificed and subcutaneous tumors were subjected to qPCR, Western blotting, and IHC staining. Similarly, 5 × 10^6^ sh-circSLC7A11 or negative control HCCLM3 cells were injected into the tail vein of each nude mouse to construct tumor metastasis models. After 8 weeks, the lungs were removed and validated using hematoxylin and eosin (H&E) staining.

### Statistical analyses

Statistical analyses were performed using SPSS (ver. 18.0; SPSS Inc., USA) and GraphPad Prism 6 software (GraphPad Software Inc., USA). Student’s *t*-test and one-way analysis of variance (ANOVA) were performed to evaluate group differences. Low and high circSLC7A11 expression levels or low and high miR-330-3p expression levels were cut off by median expression values, respectively. Association of circSLC7A11 or miR-330-3p expression level with clinicopathological parameters were evaluated by the chi-squared test or Fisher’s exact test. Pearson’s correlation was used to analyze correlations among circSLC7A11, miR-330-3p, and CDK1. Data are expressed as means ± standard deviation (SD). For all analyses, *p* < 0.05 was taken to indicate statistical significance.

## Results

### Differentially expressed circRNAs in HCC tissues and matched normal tissues

**Fig. 1:**
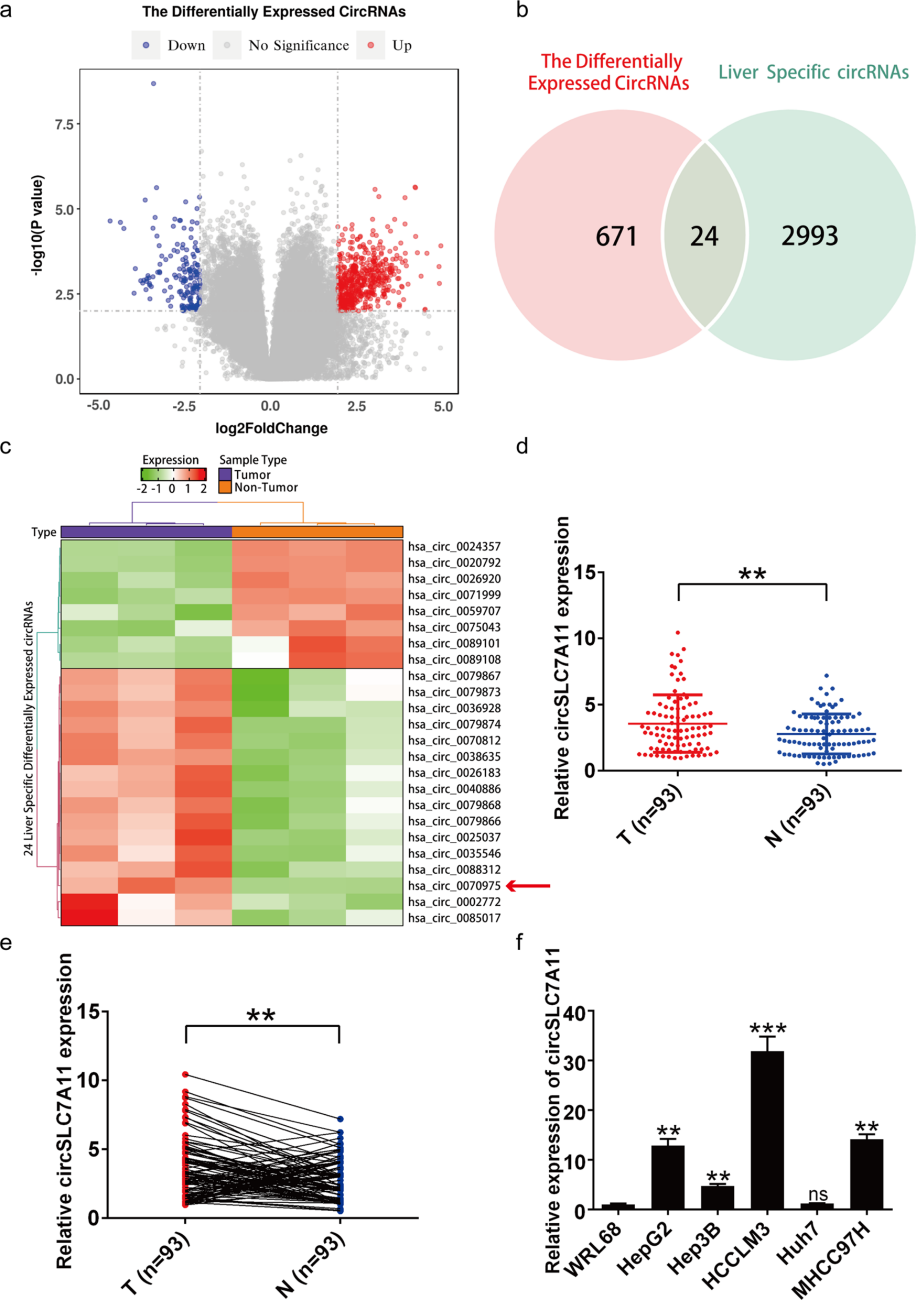
Identification of circSLC7A11 as a circRNA associated with hepatocellular carcinoma (HCC) progression. **a** Volcano plot of circRNA abundance in three HCC tissues and their paired adjacent tissues. Horizontal dashed line corresponds to *p* = 0.01. Vertical dashed lines correspond to upregulation (fold change > 4) and downregulation (fold change < –4). **b** Venn diagram showing the overlapping of differentially expressed circRNAs identified through RNA sequencing (RNA-seq) (left) and liver-specific circRNAs identified in the circBase database (right). **c** Heatmap of 24 overlapping liver-specific differentially expressed circRNAs relative to paired adjacent tissues. Rows represent circRNAs; columns represent tissues. **d**, **e** Relative expression of circSLC7A11 in HCC tissues and paired adjacent tissues detected by quantitative reverse-transcription polymerase chain reaction (qRT-PCR) T, tumor (n = 93); N, non-tumor (n = 93) (*p* = 0.0053). **f** Relative expression of circSLC7A11 in HCC cell lines relative to the immortalized hepatocyte cell line WRL68. Data are mean ± standard deviation (SD) (ns = not significant; *p < 0.05; **p < 0.01; ***p < 0.001)

To explore the role of circRNAs in the initiation and progression of HCC, we performed circRNA-seq analysis of total RNA obtained from three clinical HCC tissues and their matched adjacent tissues. We detected a total of 88,750 circRNAs in one or more of the samples. Among these, we identified 695 circRNAs that were differentially expressed (*p* < 0.01, fold change > 4) between cancer tissues and their paired adjacent tissues. Because circRNA expression levels varied among cell and tissue types, we downloaded the liver-specific circRNA profiling database, circBase [34]. Among the 695 differentially expressed circRNAs, 24 were annotated in circBase as liver-specific; among these, 16 were upregulated and 8 were downregulated in HCC tissues relative to their paired adjacent tissues (Fig. 1a and b). Hsa_circ_0070975 (termed circSLC7A11 in this study) showed the highest upregulation (fold-change = 54.61) among the 16 upregulated circRNAs in a cluster heatmap of the 24 dysregulated circRNAs (Fig. 1c). We further circSLC7A11 expression levels in 93 HCC tissues and their paired adjacent tissues. As expected, circSLC7A11 was upregulated in HCC tissues compared with the paired adjacent tissues, consistent with our RNA-seq results (Fig. 1d and e). CircSLC7A11 expression levels were also compared between the WRL68 cell line and HepG2, Hep3B, HCCLM3, Huh7, and MHCC97H cell lines (Fig. 1f). Among these cell lines, we selected the HCCLM3 and Hep3B cell lines to explore the role of the downstream regulatory pathway of circSLC7A11 in HCC progression.

## Description and clinical features of circSLC7A11

**Fig. 2:**
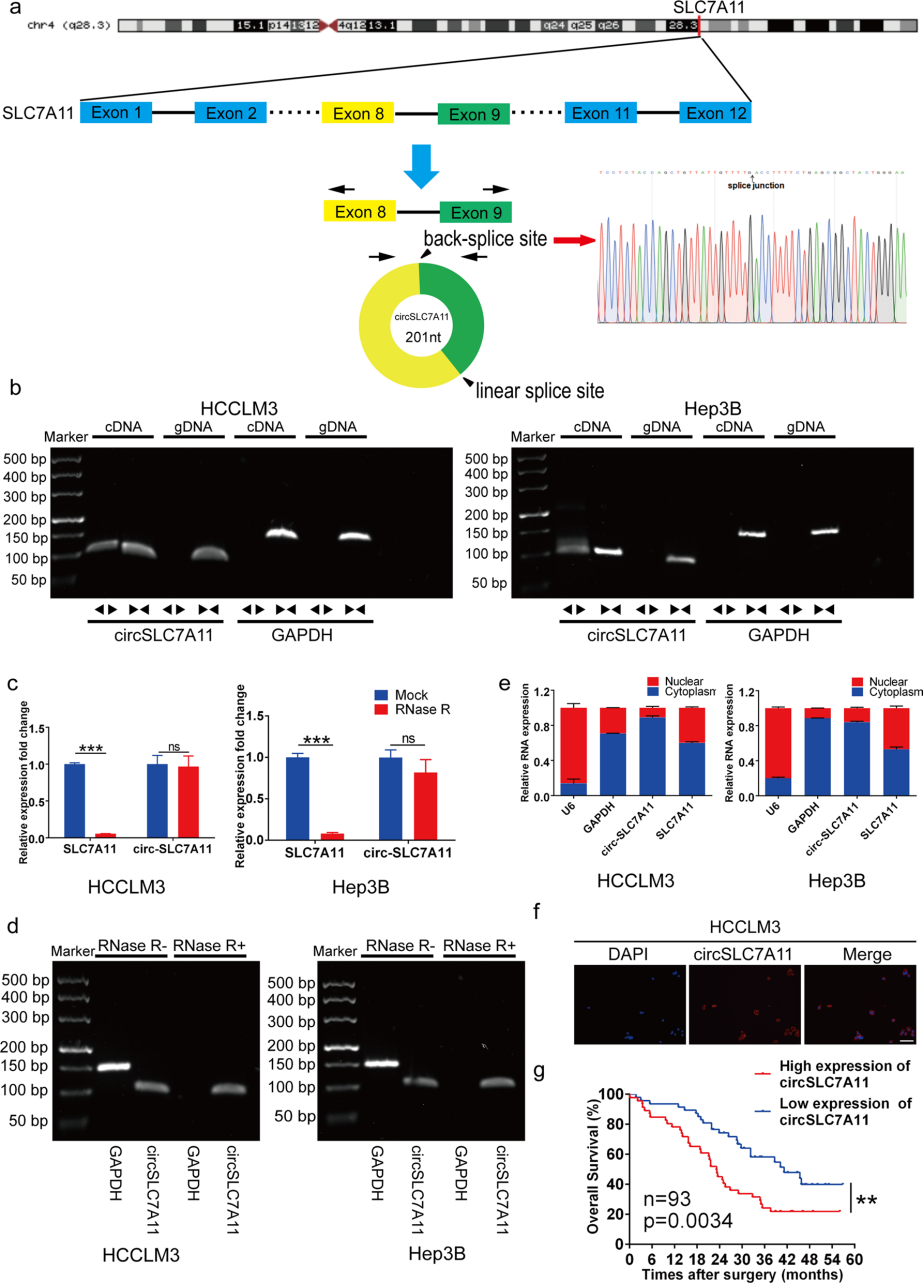
Description and clinical features of circSLC7A11. **a** Schematic illustration of circSLC7A11 and the head-to-tail splicing junction site, validated by Sanger sequencing. **b** Nucleic acid electrophoresis confirmed the presence of circSLC7A11 in HCCLM3 and Hep3B cells. CircSLC7A11 was amplified by divergent primers in cDNA, but not gDNA. GAPDH was used as a negative control. **c** Relative expression of circSLC7A11 and SLC7A11 mRNA in HCCLM3 and Hep3B cells in the presence or absence of RNase R. **d** The qRT-PCR product of circSLC7A11 was detected by nucleic acid electrophoresis in the presence or absence of RNase R. GAPDH was used as a negative control. **e** A nucleocytoplasmic fractionation assay indicated that circSLC7A11 is mainly localized in the cytoplasm of HCCLM3 and Hep3B cells. GAPDH and U6 were used as negative controls. **f** A fluorescence *in situ* hybridization (FISH) assay demonstrated the localization of circSLC7A11 in HCCLM3 and Hep3B cells. Blue indicates nuclei stained with DAPI; red indicates circSLC7A11 staining. Scale bar, 100 μm. **g** Survival curves of HCC patients with low and high expression of circSLC7A11 (n = 93, *p* = 0.0034). Data are means ± SD (ns = not significant, *p < 0.05; **p < 0.01; ***p < 0.001)

CircSLC7A11 is derived from the SLC7A11 gene, and comprises the splicing junction of exons 8 and 9 (chromosome 4, 139,103,450–139,104,459) (Fig. 2a). Because head-to-tail splicing can be caused by either trans-splicing or genomic rearrangement, we designed convergent primers and special divergent primers to amplify circSLC7A11. We subjected cDNA and gDNA obtained from HCCLM3 and Hep3B cells to qRT-PCR and nucleic acid electrophoresis; the results showed that circSLC7A11 was amplified by divergent primers in the cDNA, but not the extracted gDNA (Fig. 2b). Next, we confirmed the head-to-tail splicing junction site of circSLC7A11 through Sanger sequencing; the results were identical to those reported in circBase (Fig. 2a). Stability is a vital feature of circRNAs [35]. RNase R was used to confirm the stability of circSLC7A11 in this study, and qRT-PCR and nucleic acid electrophoresis results indicated that circSLC7A11, but not linear SLC7A11 or GAPDH, resisted digestion by RNase R (Fig. 2c and d). Thus, circSLC7A11 was detected with divergent primers only in cDNA PCR products, and not in gDNA PCR products, even under RNase R treatment. These results demonstrate that circSLC7A11 is not a by-product of genomic rearrangement. To explore the subcellular location of circSLC7A11 in HCC cells, we performed FISH and nucleocytoplasmic fractionation assays; the results revealed that circSLC7A11 is mainly located in, and thus probably mainly functions in, the cytoplasm (Fig. 2e and f).

Clinicopathological data of enrolled HCC patients revealed that circSLC7A11 expression levels were positively correlated with tumor size, microvascular invasion, and TNM stage (Table 1). Survival analysis revealed that HCC patients with high circSLC7A11 expression generally had worse prognoses (Fig. 2g). These results confirm that circSLC7A11 is a promising candidate prognostic marker for HCC.

**Table 1:** Correlation between expression of circSLC7A11 and miR-330-3p and clinicopathological features in 93 HCC patients

Clinical parameter	circSLC7A11 expression		*P* value	miR-330-3p expression		*P* value
	High	Low		High	Low	
	n = 47	n = 46		n = 47	n = 46	
Age at surgery (years)			0.470			0.574
≥ 60	31	27		28	30	
< 60	16	19		19	16	
Gender			0.114			0.894
Male	33	25		29	29	
Female	14	21		18	17	
HBV			0.353			0.078
Yes	27	22		29	20	
No	20	24		18	26	
HCV			0.725			0.17
Yes	18	16		14	20	
No	29	30		33	26	
AFP			0.354			0.747
≥ 20 µg/L	28	23		25	26	
< 20 µg/L	19	23		22	20	
Liver cirrhosis			0.768			0.065
Yes	30	28		25	33	
No	17	18		22	13	
Tumor size			**0.017**			**0.003**
≥ 5 cm	29	17		16	30	
< 5 cm	18	29		31	16	
Tumor number			0.415			**0.022**
Single	30	33		37	26	
Multiple	17	13		10	20	
Microvascular invasion			**0.009**			**0.049**
Yes	27	14		16	25	
No	20	32		31	21	
TNM stage			**0.005**			**0.027**
I–II	14	27		26	15	
III–IV	33	19		21	31	

*Statistical analyses by Pearson’s χ2 test or Fisher’s exact test

### CircSLC7A11 promoted HCC progression in vitro

**Fig. 3:**
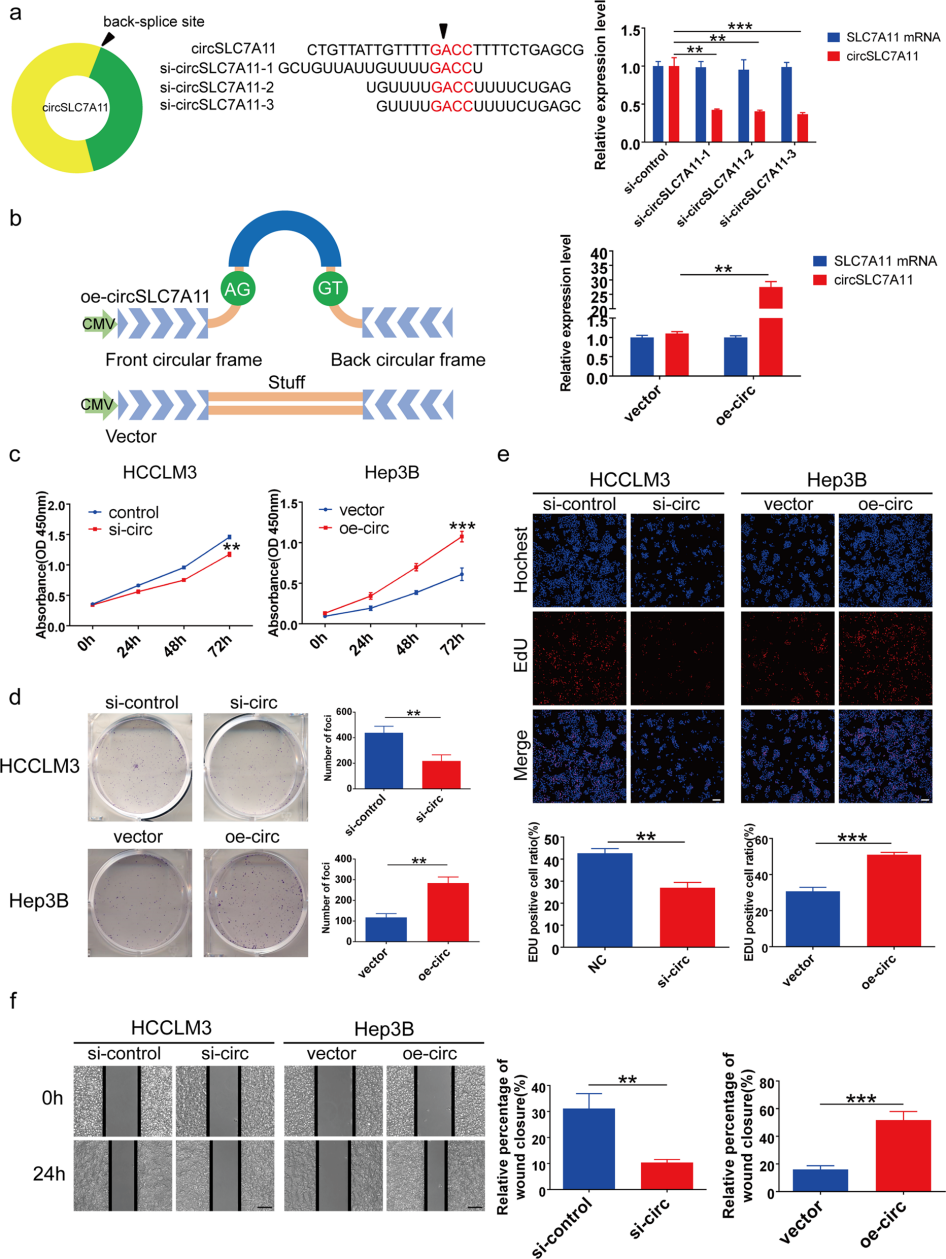
CircSLC7A11 accelerated HCC progression and metastasis ***in vitro***. **a** Schematic illustration of siRNAs and relative expression of circSLC7A11 and SLC7A11 mRNA in HCCLM3 cells treated with siRNAs. **b** Schematic illustration of circSLC7A11 expression vector and relative expression of circSLC7A11 and SLC7A11 mRNA in Hep3B cells transfected with circSLC7A11 expression vector. **c**–**e** Cell Counting Kit-8 (CCK-8), colony formation, and 5-ethynyl-20-deoxyuridine (EdU) assays revealed cell proliferation in HCCLM3 cells transfected with si-circ or negative control, and in Hep3B cells transfected with oe-circ or vector. The end point of colony formation assay in our research was 14 days. Scale bar, 100 μm. **f** Cell migration detected by wound-healing assay in HCCLM3 cells transfected with si-circ or negative control, and in Hep3B cells transfected with oe-circ or vector. Scale bar, 200 μm. Data are means ± SD (*p < 0.05; **p < 0.01; ***p < 0.001)

To investigate the function and mechanism of circSLC7A11 in HCC cells, we synthesized three siRNAs against circSLC7A11 and a circSLC7A11 overexpression vector. After transfection, circSLC7A11 expression, but not SLC7A11 mRNA expression, was suppressed in HCCLM3 cells. We selected si-circSLC7A11-3 for subsequent experiments due to its high efficiency (Fig. 3a). The circSLC7A11 overexpression vector successfully increased circSLC7A11 expression, but not SLC7A11 mRNA expression, in Hep3B cells (Fig. 3b).

CCK-8 assays showed that circSLC7A11 silencing significantly inhibited HCC cell proliferation, whereas circSLC7A11 upregulation had the opposite effect (Fig. 3c). A colony formation assay indicated that the number of HCCLM3 cell colonies was significantly decreased by circSLC7A11 silencing, and significantly increased by circSLC7A11 overexpression, in Hep3B cells (Fig. 3d) Similarly, EdU assays indicated that circSLC7A11 knockdown significantly decreased the proportion of EdU-positive cells, whereas circSLC7A11 upregulated had the opposite effect (Fig. 3e).

Wound-healing test results indicated that HCC cell migration was markedly decreased by circSLC7A11 silencing, but markedly increased by circSLC7A11 upregulation (Fig. 3f). In summary, our results demonstrated that upregulated circSLC7A11 expression promoted HCC progression and metastasis in vitro.

### MiR-330-3p downregulation was correlated with HCC clinicopathological features and prognosis

**Fig. 4:**
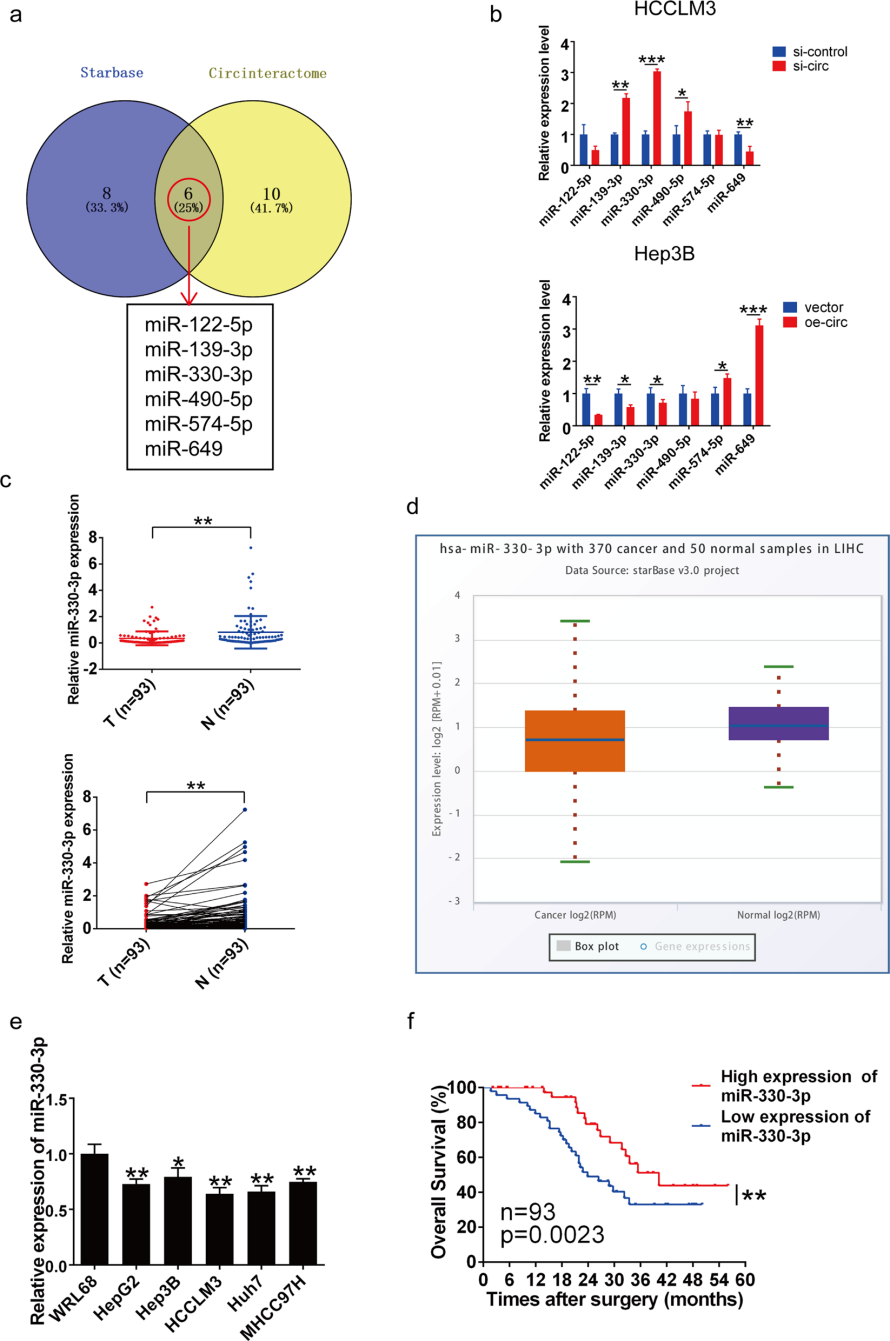
MiR-330-3p was downregulated in HCC tissues in a manner correlated with HCC prognosis. **a** Schematic illustration of overlapping potential target miRNAs of circSLC7A11, as predicted by the Starbase and Circinteractome online tools. **b** Relative expression of candidate miRNAs in transfected HCCLM3 and Hep3B cells. **c** Relative expression of miR-330-3p in HCC tissues and paired adjacent tissues T, tumor (n = 93); N, non-tumor (n = 93) (*p* = 0.001). **d** Box plot of miR-330-3p expression in The Cancer Genome Atlas (TCGA) HCC tumors and paired normal liver tissues. **e** Relative expression of miR-330-3p in HCC cell lines relative to the immortalized hepatocyte cell line WRL68. Data are means ± SD (ns = not significant, *p < 0.05; **p < 0.01; ***p < 0.001). **f** Survival curves for HCC patients showing low and high miR-330-3p expression (n = 93, *p* = 0.0023)

Based on the theory of ceRNA, circRNAs usually function as miRNA sponges efficiently regulating the expression of target genes [13, 25]. Because circSLC7A11 was mainly located in the cytoplasm, we investigated whether circSLC7A11 promoted HCC progression *in vitro* by sponging miRNAs. We predicted potential miRNA targets of circSLC7A11 using the Starbase and Circinteractome online tools. Overlapping the results of both databases, we identified six target miRNAs that could be involved in HCC initiation and progression (Fig. 4a). For further validation, we compared the expression levels of candidate miRNAs in transfected cells; the expression levels of miR-330-3p and miR-139-3p were highly upregulated in the si-circSLC7A11 group, and significantly downregulated in the circSLC7A11 overexpression group, compared with other candidate miRNAs (Fig. 4b). We also examined miR-330-3p expression in 93 HCC patients and found that miR-330-3p was downregulated in HCC tissues compared with matched adjacent normal tissues, consistent with information from The Cancer Genome Atlas (TCGA) database (Fig. 4c and d). MiR-330-3p downregulation was also detected in HCC cells compared with WRL68 cells (Fig. 4e).

The relationship between miR-330-3p expression and clinicopathological features of HCC patients is shown in Table 1. MiR-330-3p expression was negatively correlated with tumor size, tumor number, microvascular invasion, and TNM stage. Survival analysis also revealed that low miR-330-3p expression was correlated with poor prognosis in HCC patients (Fig. 4f), indicating that miR-330-3p may be a useful prognostic marker of HCC. Thus, as a potential target of circSLC7A11, miR-330-3p functioned as a tumor-suppressing factor, and its expression was correlated with the clinicopathological features and prognosis of HCC.

### MiR-330-3p suppressed HCC cell proliferation, and migration in vitro by targeting CDK1

**Fig. 5:**
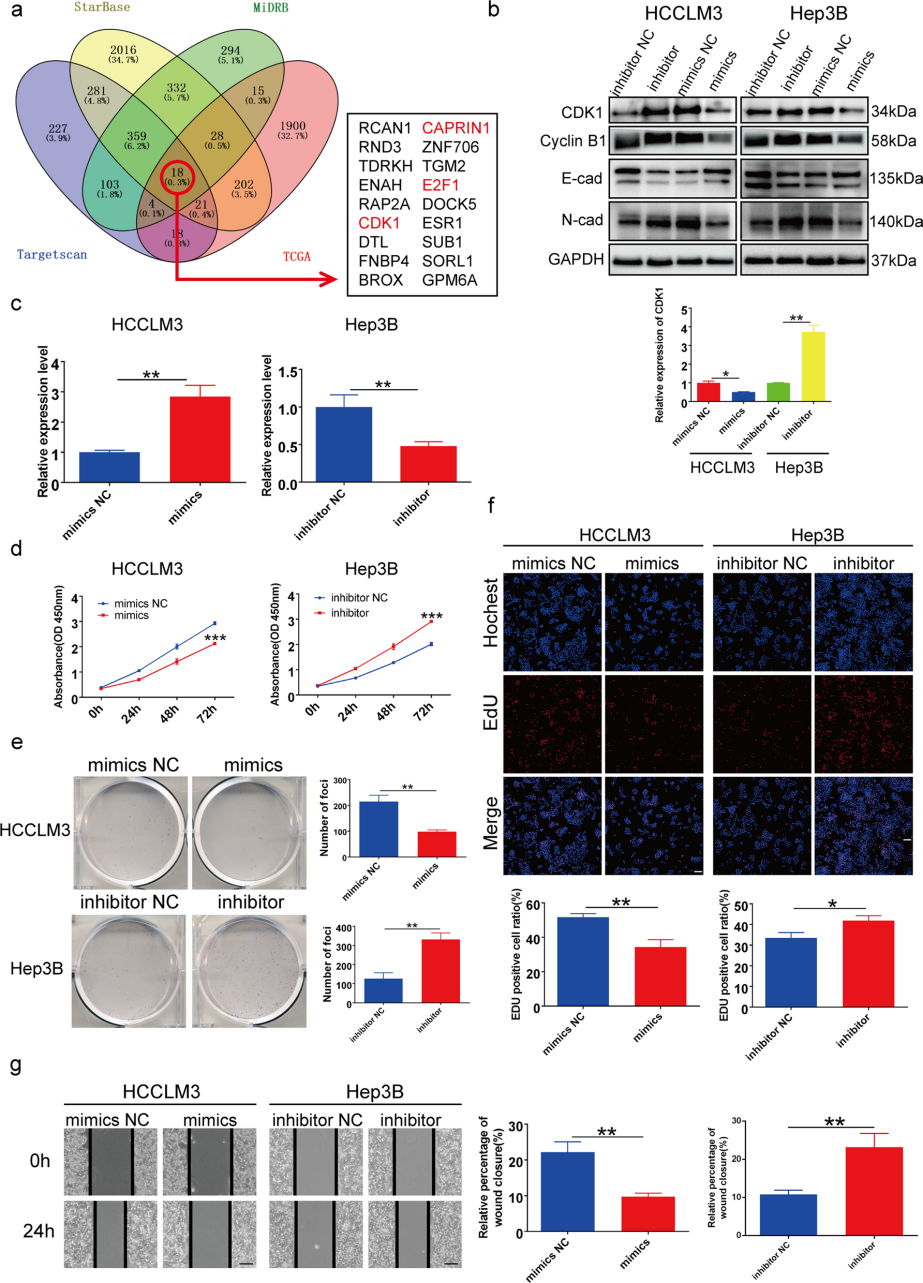
MiR-330-3p suppressed HCC cell proliferation and migration ***in vitro*** by targeting cyclin-dependent kinase 1 (CDK1). **a** Schematic illustration of target genes of miR-330-3p predicted by the Starbase, miRDB, Targetscan, and TCGA databases. **b** Relative mRNA and protein levels of CDK1 in HCCLM3 and Hep3B cells transfected with an miR-330-3p mimic or inhibitor. **c** Transfection efficiency was detected by qRT-PCR in HCCLM3 and Hep3B cells. **d**–**f** CCK-8, colony formation, and EdU assays detected the proliferation of HCCLM3 cells transfected with a mimic or mimic negative control, and Hep3B cells transfected with inhibitor or inhibitor negative control. Scale bar, 100 μm. **g** Cell migration detected by wound-healing assay in HCCLM3 cells transfected with a mimic or negative control mimic, and Hep3B cells transfected with inhibitor or inhibitor negative control. Scale bar, 200 μm. Data are means ± SD (*p < 0.05; **p < 0.01; ***p < 0.001)

miRNAs, which act as negative regulators to suppress the expression of target genes, have been found to be related to HCC initiation and progression [36]. However, few studies have explored the role of miR-330-3p in HCC; therefore, we explored the biological function and mechanism of miR-330-3p in HCC. First, we predicted the potential target genes of miR-330-3p using online target prediction tools including Starbase, MiRDB, and Targetscan. We overlapped the results with the TCGA database and identified 18 potential target genes for further validation (Fig. 5a). Among these target genes, only CDK1, CAPRIN1, and E2F1 have previously been associated with HCC progression. Next, we transfected HCC cells with an miR-330-3p inhibitor and mimic to compare the expression levels of potential target genes. The results showed that CDK1 enhanced the miR-330-3p inhibitor group and impaired the miR-330-3p mimic group, whereas the other candidate genes did not (Fig. 5b and Additional file 4: Fig. S1) Therefore, we selected CDK1 as a target gene of miR-330-3p for subsequent investigation.

We further explored the biological function of miR-330-3p in HCC cells. Based on miR-330-3p expression in HCC cell lines (Fig. 4e), Hep3B cells were transfected with an miR-330-3p inhibitor, whereas HCCLM3 cells were transfected with an miR-330-3p mimic. Transfection efficiency was verified by qRT-PCR (Fig. 5c). A CCK-8 assay revealed that increased miR-330-3p expression significantly attenuated HCC cell proliferation, whereas miR-330-3p silencing exerted the opposite effect, consistent with the results of the colony formation assay (Fig. 5d and e) Similarly, EdU assays demonstrated that miR-330-3p silencing increased the proportion of EdU-positive cells, whereas increased miR-330-3p expression clearly had the opposite effect (Fig. 5f).

The results of a wound-healing test indicated that HCC cell migration was markedly enhanced by miR-330-3p silencing but attenuated by increased miR-330-3p expression (Fig. 5g). Thus, miR-330-3p suppressed HCC cell proliferation, and migration in vitro.

### CircSLC7A11 absorbed miR-330-3p to regulate CDK1 expression

**Fig. 6:**
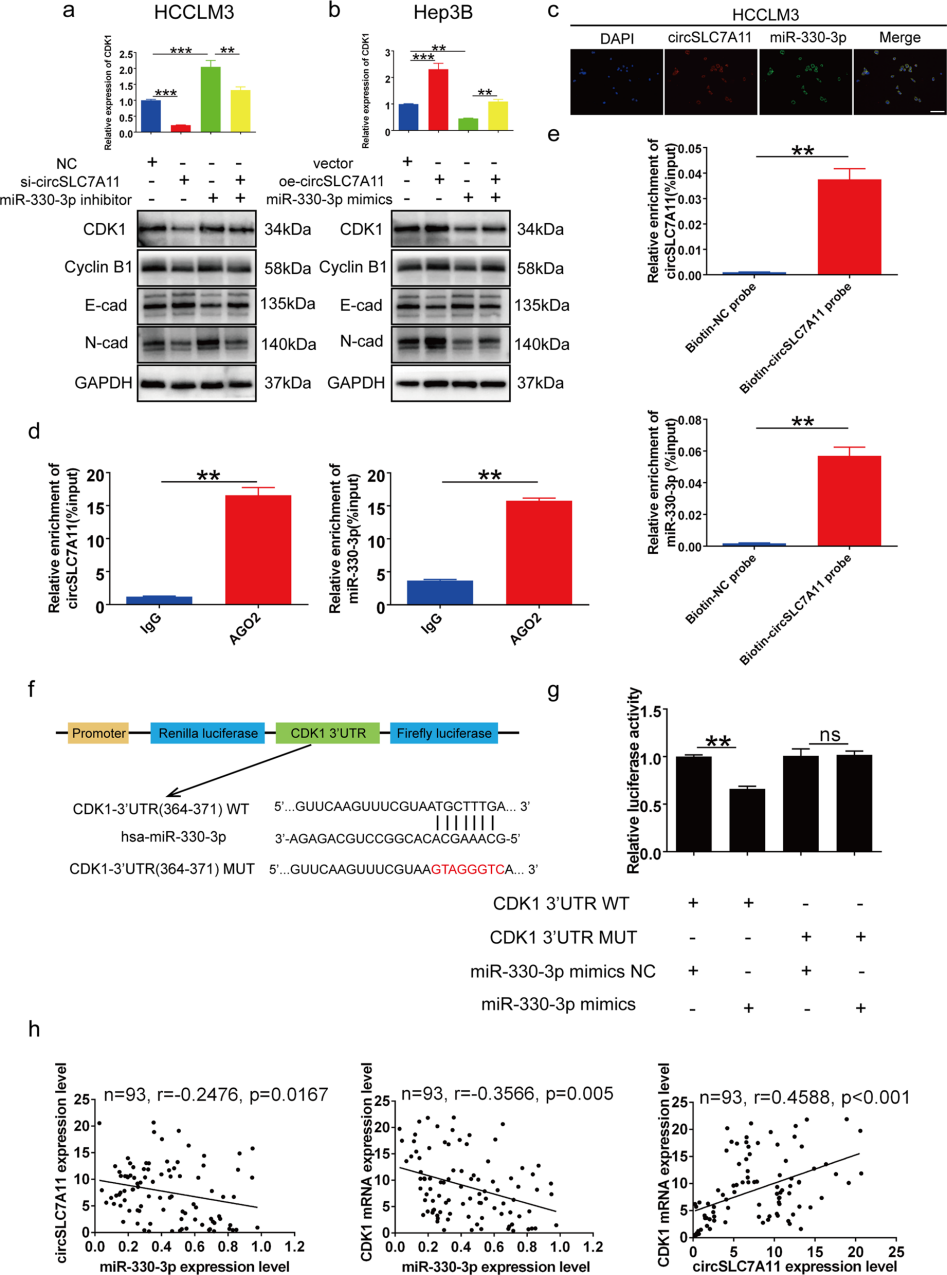
CircSLC7A11 functioned as an miR-330-3p sponge to regulate CDK1 expression. **a** Relative mRNA and protein levels of CDK1 detected in HCCLM3 cells transfected with negative control, si-circ, and miR-330-3p inhibitor according to qRT-PCR (upper) and Western blotting (bottom). **b** Relative mRNA and protein levels of CDK1 detected in Hep3B cells transfected with vector, oe-circ, and miR-330-3p mimic using qRT-PCR (upper) and Western blot (bottom). **c** A FISH assay showed the cellular localization of circSLC7A11 (red) and miR-330-3p (green) in HCC cells. Scale bar, 100 μm. **d** An RNA immunoprecipitation assay detected circSLC7A11 and miR-330-3p binding to Ago2. **e** qRT-PCR showed enrichment of circSLC7A11 and miR-330-3p in a biotin-labeled RNA pulldown assay of HCCLM3 cells. **f** Schematic illustration of CDK1-3’UTR-WT and CDK1-3’UTR-MUT luciferase reporter vectors. **g** Relative luciferase activities detected in HEK-293 T cells after co-transfection with CDK1-3’UTR-WT or CDK1-3’UTR-MUT and a mimic or negative control. **h** Pearson correlation analysis of circSLC7A11, miR-330-3p, and CDK1 expression. n = 93; Data are means ± SD (ns = not significant, *p < 0.05; **p < 0.01; ***p < 0.001)

Because circRNAs alter the expression of downstream target genes by acting as miRNA sponges, binding to proteins, and participating in protein translation [10, 11], we performed a series of experiments to further validate the interactions among circSLC7A11, miR-330-3p and CDK1. Both qRT-PCR and Western blotting indicated that circSLC7A11 silencing significantly downregulated CDK1 expression, whereas miR-330-3p inhibition enhanced CDK1 expression, and co-transfection of si-circSLC7A11 and miR-330-3p inhibitor counteracted their effects in HCCLM3 cells (Fig. 6a). Similarly, circSLC7A11 overexpression markedly increased CDK1 expression, whereas miR-330-3p mimics exerted the opposite effect, and co-transfection of oe-circSLC7A11 and miR-330-3p mimics counteracted their effects in Hep3B cells (Fig. 6b). Given that circRNAs typically act as miRNA sponges, we conducted a FISH assay to determine the subcellular locations of circSLC7A11 and miR-330-3p. As expected, circSLC7A11 and miR-330-3p were co-located in the cytoplasm (Fig. 6c). To further validate direct interaction between circSLC7A11 and miR-330-3p, we performed RIP and biotin-labeled RNA pulldown assays. As expected, the results of the RIP assay showed that both circSLC7A11 and miR-330-3p were efficiently pulled down by anti-Ago2 (*p* < 0.01), suggesting direct interaction between circSLC7A11 and miR-330-3p (Fig. 6d). The biotin-labeled pull-down assay showed that miR-330-3p was highly enriched in the circSLC7A11 probe group compared with the control probe group (*p* < 0.01) (Fig. 6e). Together, these results indicate that circSLC7A11 can bind directly to miR-330-3p. To verify the potential binding site of miR-330-3p and CDK1 predicted by the online tools, full-length CDK1-3’UTR-WT and CDK1-3’UTR-MUT were cloned into the luciferase reporter vector PGL3 and then co-transfected with an miR-330-3p mimic or negative control into HEK-293 T cells, to detect interaction between miR-330-3p and CDK1 (Fig. 6f). The results indicated that luciferase activity of CDK1-3’UTR-WT was significantly decreased in the miR-330-3p mimic group; corresponding effects were not detected in CDK1-3’UTR-MUT, suggesting direct interaction between miR-330-3p and CDK1 (Fig. 6 g). The qRT-PCR results from 93 HCC tissues showed strong negative correlations between miR-330-3p and both circSLC7A11 and CDK1, and a strong positive correlation between circSLC7A11 and CDK1 (Fig. 6 h). Together, these results indicate that circSLC7A11 acts as a sponge for miR-330-3p, thereby regulating CDK1 expression in HCC.

### CircSLC7A11 promoted HCC progression via the circSLC7A11/miR-330-3p/CDK1 axis

**Fig. 7:**
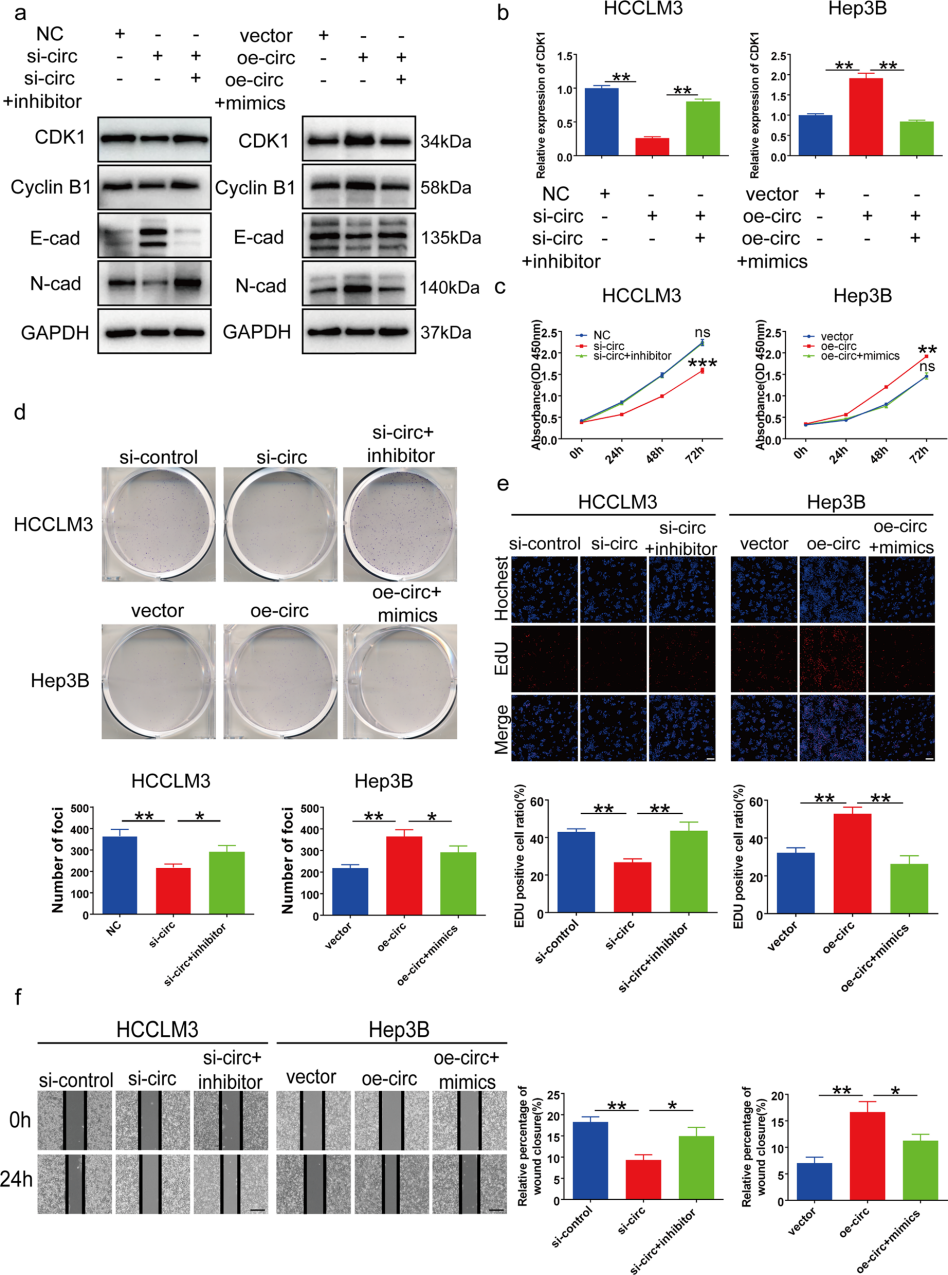
CircSLC7A11 accelerated HCC progression through the circSLC7A11/miR-330-3p/CDK1 axis. **a**, **b** Relative mRNA and protein levels of CDK1 in HCCLM3 and Hep3B cells transfected with si-circ, oe-circ, mimics, inhibitor, or negative control, detected by Western blotting and qRT-PCR. **c**–**e** CCK-8, colony formation, and EdU assays detected proliferation in HCCLM3 and Hep3B cells transfected with si-circ, oe-circ, mimics, inhibitor, or negative control. Scale bar, 100 μm. **f** Cell migration detected by wound-healing assay in HCCLM3, and Hep3B cells transfected with si-circ, oe-circ, mimics, inhibitor, or negative control. Scale bar, 200 μm. Data are means ± SD (ns = not significant, *p < 0.05; **p < 0.01; ***p < 0.001)

To confirm that circSLC7A11 promoted HCC progression and metastasis through the circSLC7A11/miR-330-3p/CDK1 signaling pathway, we performed rescue experiments. Both qRT-PCR and Western blotting results indicated that circSLC7A11 silencing reduced the mRNA and protein expression of CDK1 in HCCLM3 cells, whereas circSLC7A11 overexpression enhanced CDK1 expression in Hep3B cells. Effects induced by silencing and overexpressing circSLC7A11 were counteracted by the miR-330-3p inhibitor and mimic, respectively (Fig. 7a and b). Next, we investigated whether the function of circSLC7A11 could also be reversed by an miR-330-3p inhibitor or mimic in HCC. As expected, the miR-330-3p inhibitor counteracted the suppression of proliferation and metastasis induced by circSLC7A11 silencing in HCCLM3 cells. Similarly, the miR-330-3p mimic reversed the promoting effects induced by overexpressed circSLC7A11 in Hep3B cells (Fig. 7c–f). Thus, circSLC7A11 functioned as a ceRNA by absorbing miR-330-3p to regulate CDK1 expression, resulting in HCC progression and metastasis.

### CircSLC7A11 knockdown suppressed HCC progression and lung metastasis in vivo

**Fig. 8:**
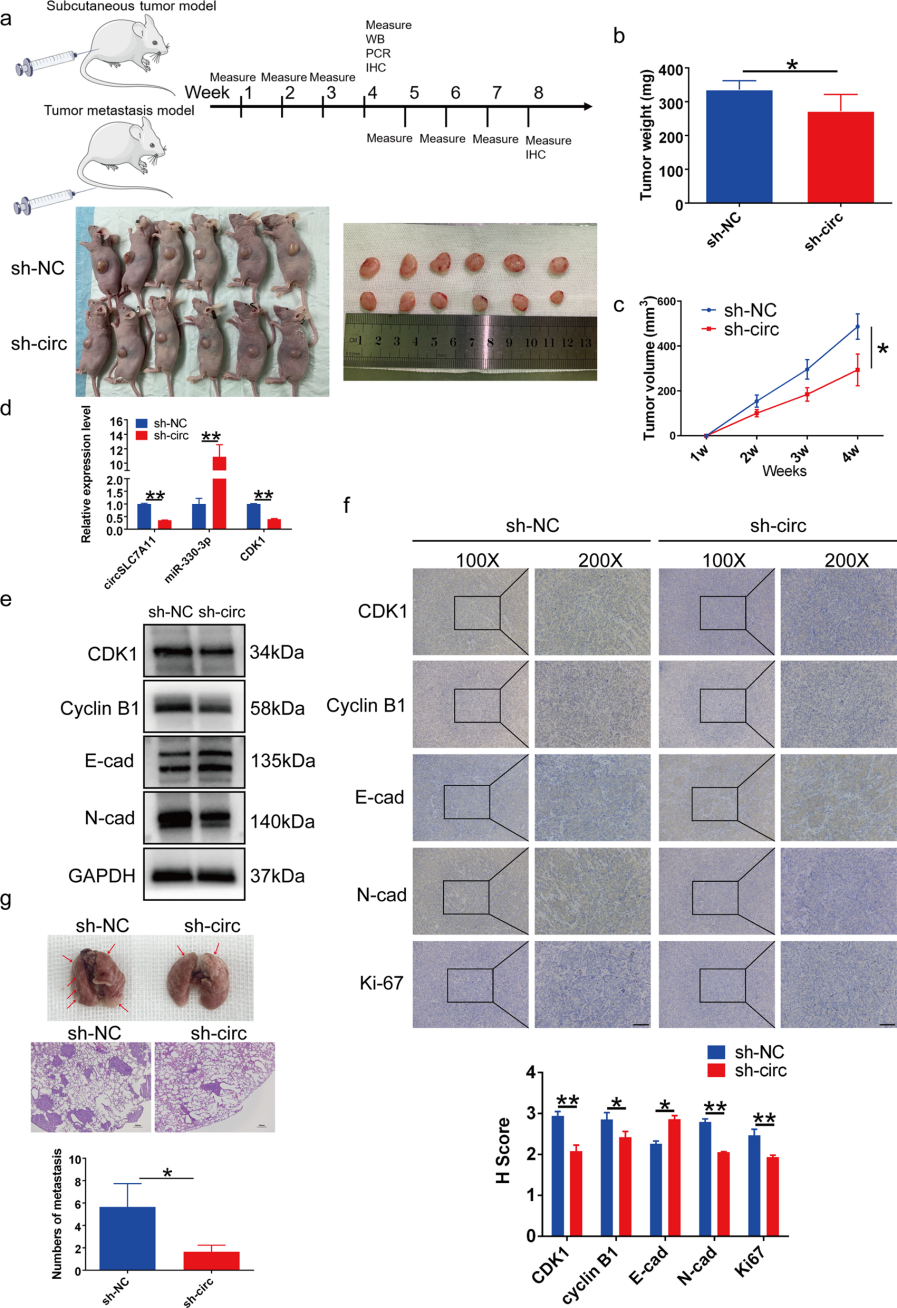
CircSLC7A11 knockdown inhibited HCC progression and metastasis in vivo. **a** Image of subcutaneous tumor tissues in the sh-circSLC7A11 and sh-NC groups (n = 6). **b** Relative tumor weights (n = 6). **c** Tumor volume was measured weekly (n = 6). **d** Relative expression levels of circSLC7A11, miR-330-3p, and CDK1 mRNA in subcutaneous tumor tissues, detected by qRT-PCR. **e** Relative expression levels of CDK1, cyclin B1, E-cadherin, and N-cadherin in subcutaneous tumor tissues, detected by Western blotting. GAPDH was used as a negative control. **f** Relative expression levels of CDK1, cyclin B1, E-cadherin, N-cadherin, and Ki-67 in subcutaneous tumor tissues, detected by immunohistochemical (IHC) analysis. **g** Image of lung metastasis in the sh-circSLC7A11 and sh-NC groups. Representative images of hematoxylin and eosin (H&E) staining of lung metastasis in HCCLM3 cells. Scale bar, 100 μm. Data are means ± SD (*p < 0.05; **p < 0.01)

**Fig. 9:**
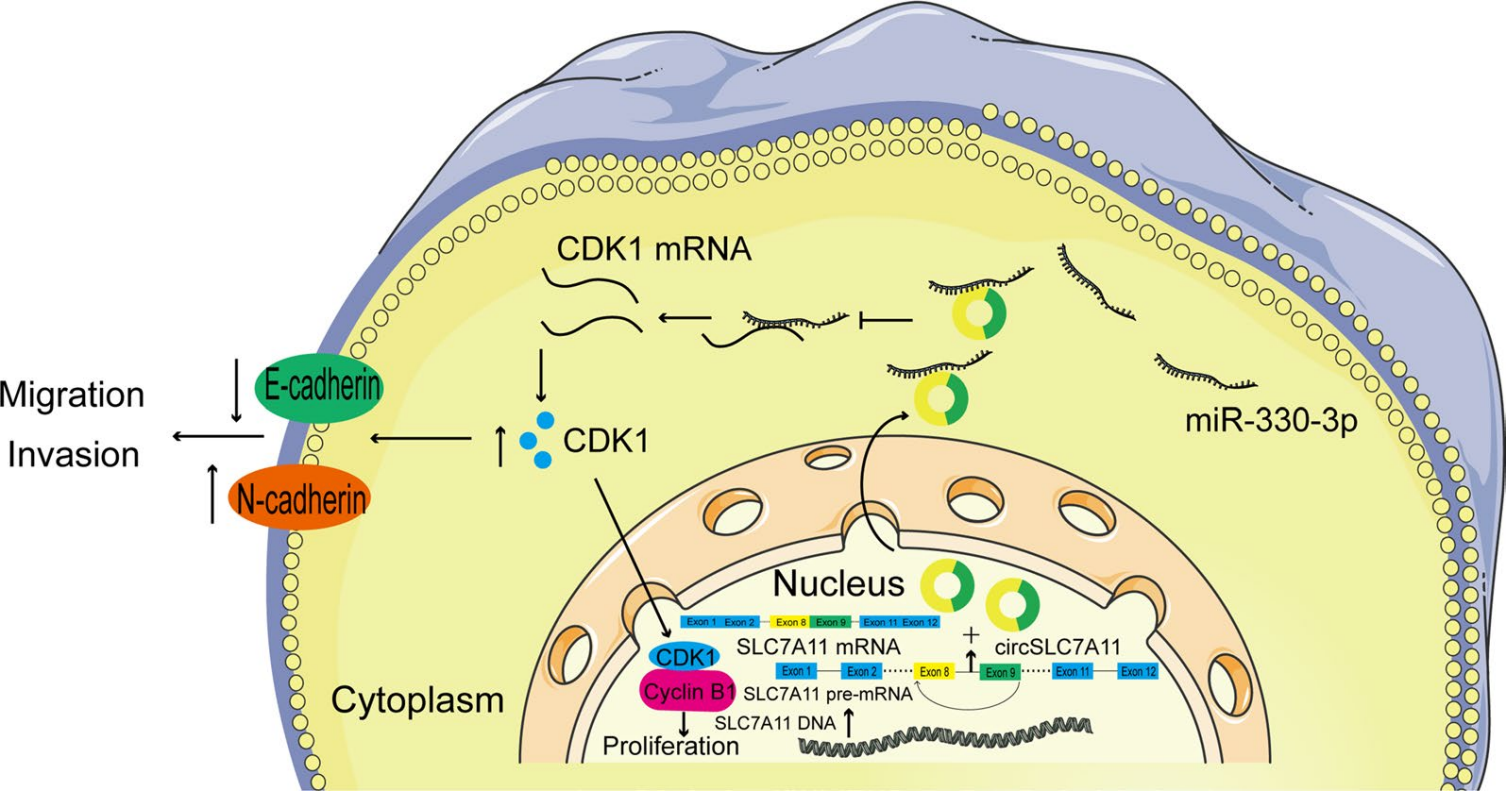
Schematic illustration of the underlying mechanism of circ SLC7A11 in HCC progression and metastasis. Schematic illustration of the underlying mechanism by which circSLC7A11 absorbs miR-330-3p to regulate CDK1 expression, and thus HCC progression and metastasis

To further investigate the function of circSLC7A11 in tumor progression in vivo, we subcutaneously injected HCCLM3 cells stably transfected with sh-circSLC7A11 or negative control into BALB/c nude mice. Transfection efficiency was confirmed using the DMi8 microscope (Additional file 4: Fig. S2). The tumor volume was measured weekly; after 4 weeks, the tumor weight of each mouse was determined. Tumor volume and weight were significantly lower in the sh-circSLC7A11 than negative control group (Fig. 8a–c). Subcutaneous tumor tissues were subjected to qRT-PCR, Western blotting, and IHC detection. The results indicated that CDK1 expression was downregulated, whereas miR-330-3p was upregulated, in the sh-circSLC7A11 group (Fig. 8d and e). IHC showed that circSLC7A11 knockdown attenuated the expression of CDK1, cyclin B1, and N-cadherin, but enhanced that of E-cadherin in xenograft tumor tissues, consistent with the *in vitro* results (Fig. 8f). To further explore the role of circSLC7A11 in tumor metastasis, we injected HCCLM3 cells stably transfected with sh-circSLC7A11 or negative control into the caudal vein of nude mice. As expected, the sh-circSLC7A11 group had fewer, smaller lung metastatic nodules than the negative control group (Fig. 8 g). Together, these findings provide the first evidence that circSLC7A11 absorbs miR-330-3p to regulate CDK1 expression, thereby promoting HCC progression *in vivo* (Fig. 9).

## Discussion

Because early stage HCC patients have no symptoms, many patients are diagnosed too late for surgery. Despite advancements in liver resection and transplantation, patients who qualify for surgery continue to be at high risk of recurrence and metastasis. Other non-surgical interventions, including TACE and RFA, provide disease control only in advanced-stage patients. Systematic administration of sorafenib or molecular targeted therapy is limited by poor efficacy and adverse effects [37]. Therefore, the development of new life-prolonging therapies for HCC patients is urgently needed.

CircRNAs are recently identified non-coding RNAs previously thought to be RNA splicing error by-products. Advances in high-throughput sequencing and bioinformatic analysis have revealed that circRNAs are ubiquitous among multiple tissues and cells, prompting widespread interest in determining the biogenesis and function of circRNAs [12]. Accumulating data on the biogenesis and function of circRNAs has provided insight into the pathogenesis of multiple diseases, such as cardiovascular disease, autoimmune disease, and cancer [38, 39]. Increasing numbers of circRNAs have been found to act as oncogenes or tumor suppressor genes in diverse cancers, including colorectal cancer, lung cancer, and GC [40–42]. To date, only a few circRNAs have been associated with HCC. In the present study, we present the first evidence that the circSLC7A11/miR-330-3p/CDK1 axis participates in HCC progression and metastasis.

We used circRNA-seq to screen differentially expressed circRNAs and discovered a novel HCC-related circRNA, circSLC7A11, which was markedly overexpressed in HCC and correlated with tumor size, microvascular invasion, TNM stage, and overall survival in HCC patients. We performed gain- and loss-of-function studies *in vivo* and *in vitro*, and found that circSLC7A11 upregulation promoted HCC proliferation and metastasis. Recent studies have revealed that circRNAs contain potential MREs that act as miRNA sponges, forming a circRNA–miRNA–mRNA axis to exert biological functions [13, 43]. Nevertheless, most circRNAs are expressed at low levels in mammals, such that they cannot effectively exert regulatory roles by absorbing miRNAs [44]. The present study showed that circSLC7A11 is highly expressed in HCC and, notably, is located in the cytoplasm of HCC cells, which prompted us to explore the ability of circSLC7A11 to act as a miRNA sponge in HCC. M^6^A is among the most common base modifications of RNA, usually occurring in the ‘RRm^6^ACH’ (R = G or A; H = A, C, or U) consensus motif [45]. Therefore, circSLC7A11 upregulation may occur due to its harbored m^6^A sites (Additional file 4: Fig. S3); we are currently exploring this potential upregulation mechanism in an ongoing study (data not shown). Because circSLC7A11 is predominantly located in the cytoplasm and contains MREs according to the Starbase and Circinteractome databases, it has been assumed to harbor miRNA binding sites for miR-330-3p, which has been reported as a tumor suppressor in some cancers including glioma, colorectal cancer, and GC [46–48]. In the present study, miR-330-3p was found to be downregulated in HCC cells and tissues, in a manner correlated with tumor size, tumor number, microvascular invasion, TNM stage, and overall survival in HCC patients. To verify the relationship between circSLC7A11 and miR-330-3p, we performed a series of experiments including FISH, RIP, and biotin-labeled probe pull-down assays, which confirmed that circSLC7A11 and miR-330-3p were co-localized and directly bound to each other in the cytoplasm. Rescue experiments further showed that miR-330-3p reversed the oncogenic function of circSLC7A11. We observed that miR-330-3p overexpression restrained CDK1 expression, suggesting that CDK1 is a direct target of miR-330-3p, which we confirmed by dual luciferase reporter assays. CDK1 is a cell cycle regulatory checkpoint that has been reported to contribute to various human malignancies. CDK1/cyclin B1 complex formation has been demonstrated to mediate mitochondrial activity during cell cycle progression and stress responses, as well as to reprogram energy metabolism in adaptive tumor resistance [49]. A previous study reported that a CDK1 inhibitor increased the efficacy of sorafenib in HCC models [50]. However, CDK1 was also demonstrated to be involved in apoptin-induced apoptosis in HCC; this tumor-suppressing role of CDK1 is inconsistent with previous findings [28]. Whether and how CDK1 mediates HCC progression remains unknown; therefore, further studies are imperative for determining the role of CDK1 in tumor progression. The results of this study confirmed that upregulated circSLC7A11 functioned as a sponge of miR-330-3p, thus neutralizing its suppressive effect on CDK1 and thereby accelerating HCC progression and metastasis; thus, it is a promising biomarker and therapeutic target for HCC patients. With regard to the growth suppression, there may be also other mechanism involved in circSLC7A11-miR-330-3p axis not limited to CDK1, which is worthy of a deeper exploration, but we believe is a result of the expression change of CDK1 to a large extent.

Recently, interactions between tumor cells and the tumor microenvironment have attracted attention worldwide. Within the tumor microenvironment, non-malignant cells coexist and interact with tumor cells through complex pathways [51]. In our ongoing research, we recently explored HCC cellular interactions in the tumor microenvironment and discovered that elevated circSLC7A11 expression in the cytoplasm of HCC cells could be transmitted to natural killer (NK) cells via exosomes (Additional file 4: Fig. S4), thus inducing NK cells exhaustion that, in turn, induces immune escape of HCC cells (data not shown).

In summary, our study provides the first data on the expression, function and clinical implications of circSLC7A11 in HCC. This is also the first study to investigate the relationships among circSLC7A11, miR-330-3p, and CDK1. Our findings provide insight into the diagnosis and treatment of HCC, despite some limitations. First, the tissues subjected to RNA-seq were obtained from a homogenous population in our hospital, and therefore do not represent all circRNAs involved in HCC progression. Second, our study demonstrated that circSLC7A11 acted as an miR-330-3p sponge, which may not be the only mechanism by which miRNA binds to circSLC7A11 to regulate HCC progression. Therefore, the diagnostic and therapeutic potential of circSLC7A11 requires further exploration.

## Conclusions

Our results demonstrated that circSLC7A11 was upregulated in HCC and functioned as an oncogene. Therefore, circSLC7A11 was shown to be a potential prognostic biomarker for HCC. We demonstrated that circSLC7A11 exerted a regulatory role on CDK1 expression through miR-330-3p absorption, thereby accelerating HCC progression and exhibiting potential as a novel therapeutic target for HCC.

## Supplementary Information

**Additional file 1: Table S1.** Primers used in this study.

**Additional file 2: Table S2.** Oligonucleotides and probes used in this study.

**Additional file 3: Table S3.** Antibodies used in this study.

**Additional file 4.** Additional figures.

## Data Availability

All data generated or analysed during this study are included in this published article and its supplementary information files.

## Abbreviations

3′UTR: 3′-untranslated region
CDK1: Cyclin-dependent kinase 1
ceRNA: Competing endogenous RNA
CircRNA: Circular RNA
FISH: Fluorescence *in situ* hybridization
gDNA: Genomic DNA
HCC: Hepatocellular carcinoma
IHC: Immunohistochemistry
miRNA: MicroRNA
MRE: miRNA response element
qRT-PCR: Quantitative real-time PCR
RBP: RNA-binding protein
RFA: Radiofrequency ablation
RIP: RNA immunoprecipitation
TACE: Trans-arterial chemoembolization
TCGA: The Cancer Genome Atlas
