# Supplementary material for: The circular RNA circSLC7A11 functions as a mir-330-3p sponge to accelerate hepatocellular carcinoma progression by regulating cyclin-dependent kinase 1 expression
Source: Cancer Cell Int. 2021 Nov 29;21:636. doi: 10.1186/s12935-021-02351-7 (PMC8628421; doi:10.1186/s12935-021-02351-7)
Supplement: Supplementary file 1 — Additional file 1: Table S1. Primers used in this study. [file 12935_2021_2351_MOESM1_ESM.docx]

Table S1 Primers used in this study

| Name | Primer | Sequence(5’-3’) |
| --- | --- | --- |
| circSLC7A11 | Forward primer | TTGTTTTGACCTTTTCTGAGC |
|  | Reverse primer | AACACACCACCGTTCATGG |
| Linear SLC7A11 | Forward primer | TCTCCAAAGGAGGTTACCTGC |
|  | Reverse primer | AGACTCCCCTCAGTAAAGTGAC |
| GAPDH | Forward primer | GGAGCGAGATCCCTCCAAAAT |
|  | Reverse primer | GGCTGTTGTCATACTTCTCATGG |
| miR-139-3p | Stem-loop Primer | CTCAACTGGTGTCGTGGAGTCGGCAATTCAGTTGAGACTCCAAC |
|  | Forward primer | ACACTCCAGCTGGGTGGAGACGCGGCCCTGT |
|  | Reverse primer | TGGTGTCGTGGAGTCG |
| miR-122-5p | Stem-loop Primer | CTCAACTGGTGTCGTGGAGTCGGCAATTCAGTTGAGCAAACACC |
|  | Forward primer | ACACTCCAGCTGGGTGGAGTGTGACAATGG |
|  | Reverse primer | TGGTGTCGTGGAGTCG |
| miR-330-3p | Stem-loop Primer | CTCAACTGGTGTCGTGGAGTCGGCAATTCAGTTGAGTCTCTGCA |
|  | Forward primer | ACACTCCAGCTGGGGCAAAGCACACGGCCTG |
|  | Reverse primer | TGGTGTCGTGGAGTCG |
| miR-490-5p | Stem-loop Primer | CTCAACTGGTGTCGTGGAGTCGGCAATTCAGTTGAGACCCACCT |
|  | Forward primer | ACACTCCAGCTGGGCCATGGATCTCCAG |
|  | Reverse primer | TGGTGTCGTGGAGTCG |
| miR-574-5p | Stem-loop Primer | CTCAACTGGTGTCGTGGAGTCGGCAATTCAGTTGAGACACACTC |
|  | Forward primer | ACACTCCAGCTGGGTGAGTGTGTGTGTGTGA |
|  | Reverse primer | TGGTGTCGTGGAGTCG |
| miR-649 | Stem-loop Primer | CTCAACTGGTGTCGTGGAGTCGGCAATTCAGTTGAGGACTCTTG |
|  | Forward primer | ACACTCCAGCTGGGAAACCTGTGTTGTTCA |
|  | Reverse primer | TGGTGTCGTGGAGTCG |
| U6 | Forward primer | CTCGCTTCGGCAGCACA |
|  | Reverse primer | AACGCTTCACGAATTTGCGT |
| CDK1 | Forward primer | AAACTACAGGTCAAGTGGTAGCC |
|  | Reverse primer | TCCTGCATAAGCACATCCTGA |
| E2F1 | Forward primer | CATCCCAGGAGGTCACTTCTG |
|  | Reverse primer | GACAACAGCGGTTCTTGCTC |
| FNBP4 | Forward primer | TTGGTGCTTATGCTGACAGTG |
|  | Reverse primer | GATCTCCGCTAGGAAGTTGGC |
| CAPRIN1 | Forward primer | GGAACGAATGAACAAAGGGGA |
|  | Reverse primer | TGACTTAGTGCCATGAAACTCCT |
| SUB1 | Forward primer | GGTGAGACTTCGAGAGCCCT |
|  | Reverse primer | GCGAACACTAACGTACCTCATTT |
| ZNF706 | Forward primer | ACAAGGACATGACCAAAAGGC |
|  | Reverse primer | GGAAGTGGAGTCTTAGGATGCTT |
| RAP2A | Forward primer | CGTGAAGCGGTATGAGAAAGT |
|  | Reverse primer | GCCTTCGCTGGACGATACT |
| ENAH | Forward primer | ACAGGTGTATGGTCTCAACTTTG |
|  | Reverse primer | TGTGAGTTTTGTCTAGGCAATGT |
| DTL | Forward primer | TGGTCTTCACAATACCCTCTTCA |
|  | Reverse primer | CTTCATTGGCAACTGCTAGTACA |
