# Supplementary material for: The circular RNA circSLC7A11 functions as a mir-330-3p sponge to accelerate hepatocellular carcinoma progression by regulating cyclin-dependent kinase 1 expression
Source: Cancer Cell Int. 2021 Nov 29;21:636. doi: 10.1186/s12935-021-02351-7 (PMC8628421; doi:10.1186/s12935-021-02351-7)
Supplement: Supplementary file 2 — Additional file 2: Table S2. Oligonucleotides and probes used in this study. [file 12935_2021_2351_MOESM2_ESM.docx]

Table S2 Oligonucleotides and probes used in this study

| Definition | Sequence (5’-3’) |
| --- | --- |
| si-circSLC7A11-NC | GGCTCTAGAAAAGCCTATGC |
| si-circSLC7A11-1 | GCUGUUAUUGUUUUGACCU |
| si-circSLC7A11-2 | UGUUUUGACCUUUUCUGAG |
| si-circSLC7A11-3 | GUUUUGACCUUUUCUGAGC |
| miR-330-3p mimics NC | Sense: UUCUCCGAACGUGUCACGUTT  Anti-sense: ACGUGACACGUUCGGAGAATT |
| miR-330-3p mimics | Sense: GCAAAGCACACGGCCUGCAGAGA  Anti-sense: UCUGCAGGCCGUGUGCUUUGCUU |
| miR-330-3p inhibitor NC | CAGUACUUUUGUGUAGUACAA |
| miR-330-3p inhibitor | UCUCUGCAGGCCGUGUGCUUUGC |
| Biotin-circSLC7A11 probe | CGCTCAGAAAAGGTCAAAACAATAACAG |
| Oligo probe | GGCTCTAGAAAAGCCTATGC |
| Cy3-labeled circSLC7A11 probe | CCGCTCAGAAAAGGTCAAAACAATAACAGC |
| Digoxin-labeled miR-330-3p probe | TCTCTGCAGGCCGTGTGCTTTGC |
