# Supplementary material for: The circular RNA circSLC7A11 functions as a mir-330-3p sponge to accelerate hepatocellular carcinoma progression by regulating cyclin-dependent kinase 1 expression
Source: Cancer Cell Int. 2021 Nov 29;21:636. doi: 10.1186/s12935-021-02351-7 (PMC8628421; doi:10.1186/s12935-021-02351-7)
Supplement: Supplementary file 3 — Additional file 3: Table S3. Antibodies used in this study. [file 12935_2021_2351_MOESM3_ESM.docx]

Table S3 Antibodies used in this study

| Antibody | Dilution of antibodies | Source | Identifier |
| --- | --- | --- | --- |
| Anti-GAPDH (WB) | 1:2000 | Proteintech | 60004-1-Ig |
| Anti-CDK1 (WB) | 1:1000 | Proteintech | 19532-1-AP |
| Anti-cyclin B1 (WB) | 1:1000 | Abcam | Ab32053 |
| Anti-E-cadherin (WB) | 1:2000 | Proteintech | 20874-1-AP |
| Anti-N-cadherin (WB) | 1:2000 | Proteintech | 22018-1-AP |
| Anti-CDK1 (IHC) | 1:400 | Proteintech | 19532-1-AP |
| Anti-cyclin B1 (IHC) | 1:250 | Abcam | Ab32053 |
| Anti-E-cadherin (IHC) | 1:400 | Proteintech | 20874-1-AP |
| Anti-N-cadherin (IHC) | 1:1000 | Proteintech | 22018-1-AP |
| Anti-Ki67 (IHC) | 1:2000 | Proteintech | 27309-1-AP |
| Anti-Ago2 (RIP) | 1:50 | Abcam | Ab186733 |
