# Supplementary material for: The circular RNA circSLC7A11 functions as a mir-330-3p sponge to accelerate hepatocellular carcinoma progression by regulating cyclin-dependent kinase 1 expression
Source: Cancer Cell Int. 2021 Nov 29;21:636. doi: 10.1186/s12935-021-02351-7 (PMC8628421; doi:10.1186/s12935-021-02351-7)
Supplement: Supplementary file 4 — Additional file 4. Additional figures. [file 12935_2021_2351_MOESM4_ESM.docx]

**
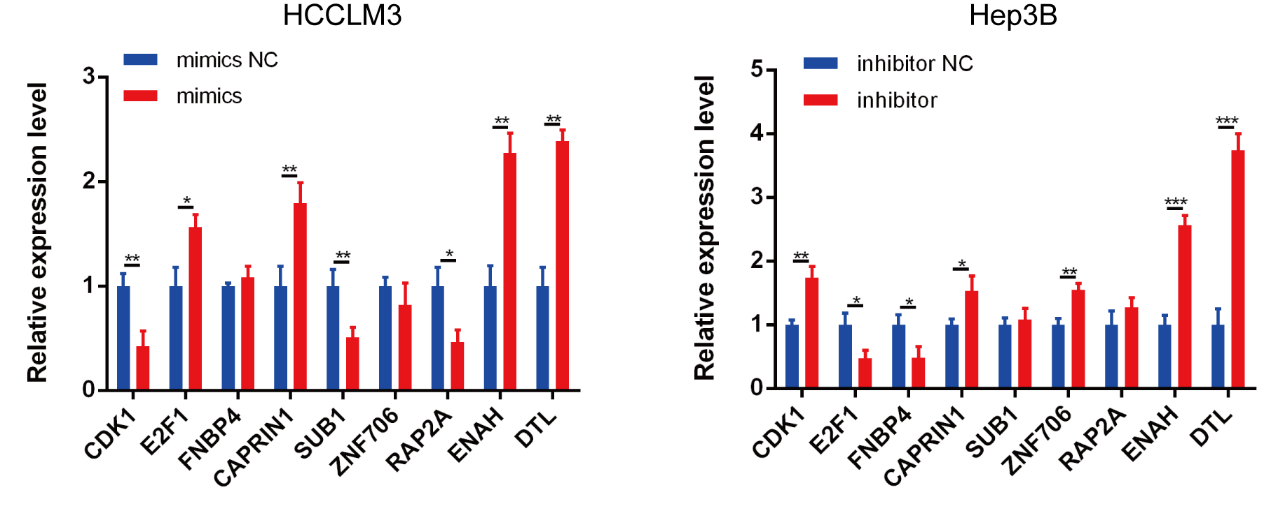
**

**Fig. S1** Relative potential target gene expression levels of miR-330-3p, evaluated by quantitative reverse-transcribed polymerase chain reaction (qRT-PCR), in HCCLM3 and Hep3B cells transfected with miR-330-3p mimic or inhibitor.

**
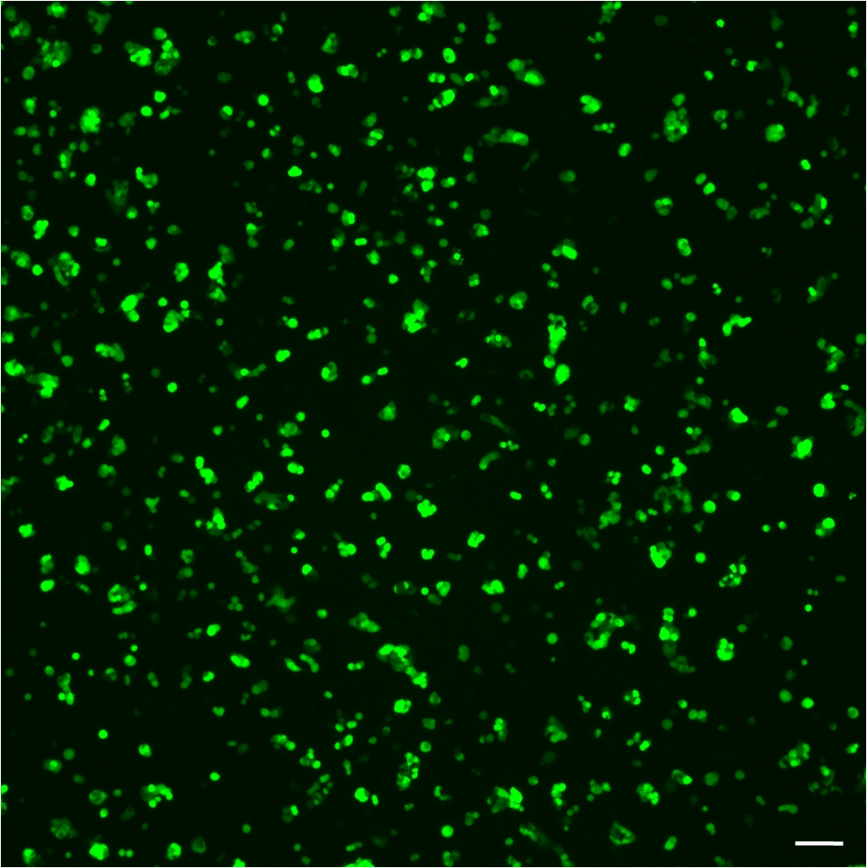
**

**Fig. S2** Stable transfection efficiency of sh-circSLC7A11 vector detected in HCCLM3 cells. Scale bar, 100 μm.


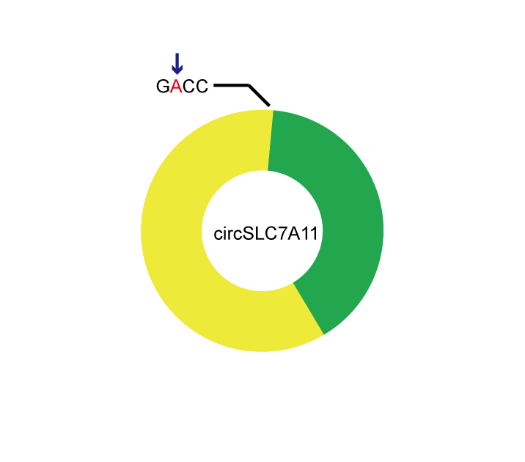


**Fig. S3** Predicted m^6^A site near the junction of circSLC7A11.


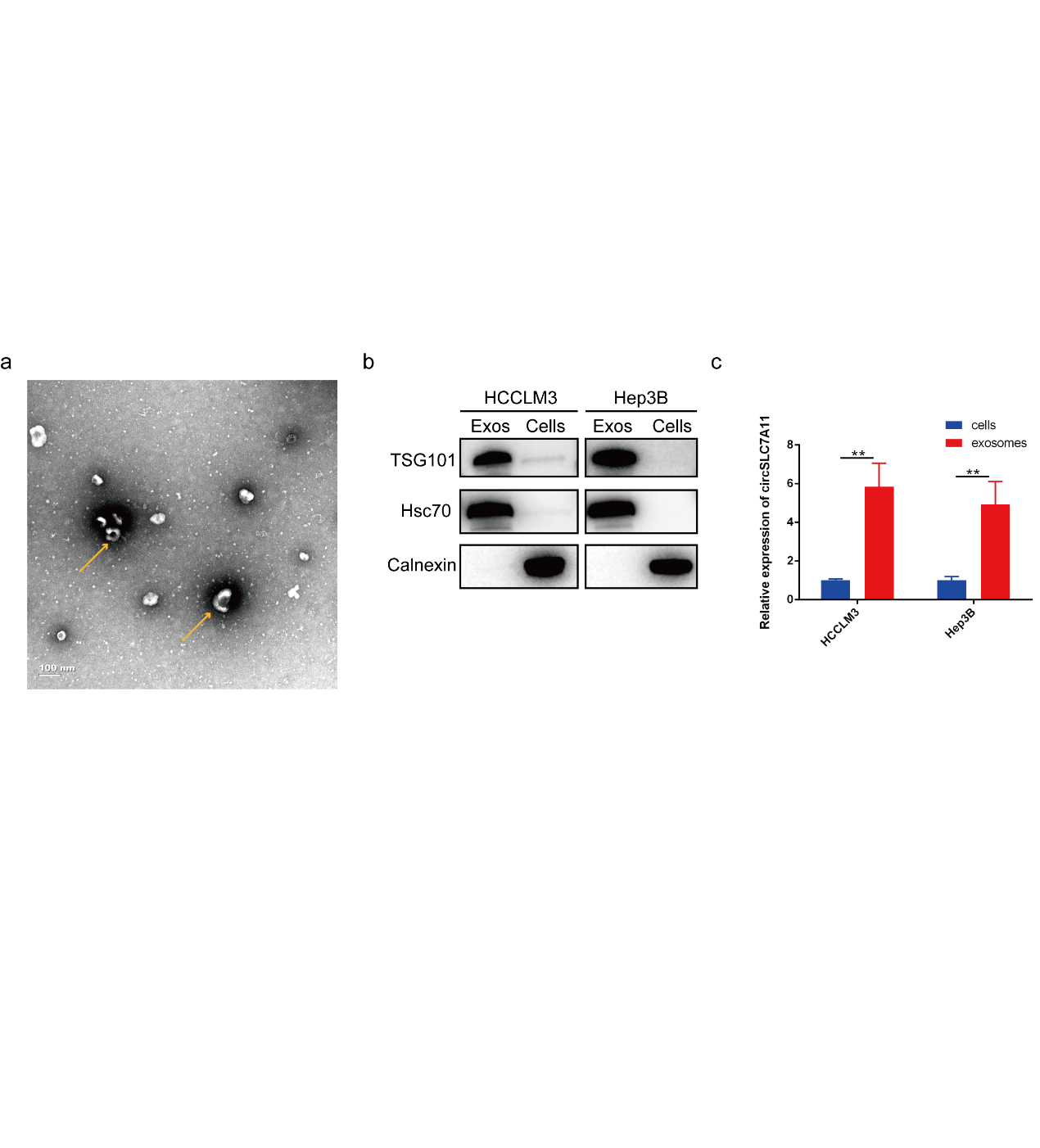


**Fig. S4** (a) Transmission electron microscopy (TEM)-based characterization of exosomes secreted by HCCLM3 cells. Scale bar, 100 nm. (b) Western blotting results revealed expression levels typical of exosomal markers. (c) Relative expression levels of circSLC7A11 in exosomes and parental cells.
